# Unfavorable course in pregnancy-associated thrombotic thrombocytopenic purpura necessitating a perimortem Cesarean section: a case report

**DOI:** 10.1186/1752-1947-7-119

**Published:** 2013-04-29

**Authors:** Ernesto González-Mesa, Isidoro Narbona, Marta Blasco, Isaac Cohen

**Affiliations:** 1 Obstetrics and Gynecology Department, Obstetrics and Gynecology Research Group. IBIMA, University Carlos Haya Hospital, Arroyo de Los Angeles Avenue, Málaga, 29011, Spain

**Keywords:** Thrombotic thrombocytopenic purpura, PTT, Perimortem Caesarean section, Microangiopathy, Maternal mortality

## Abstract

**Introduction:**

Thrombotic thrombocytopenic purpura is a type of occlusive thrombotic microangiopathy that is not specific to pregnancy but occurs with an increased frequency during it. Prognosis of thrombotic thrombocytopenic purpura greatly depends on early diagnosis and treatment. As delivery does not generally cause resolution of thrombotic thrombocytopenic purpura, pregnancy termination is not initially considered, especially under 34 weeks, although it may be required under some conditions such as preeclampsia. Plasma therapy, including plasmapheresis, and steroids are used for treatment. In the event of an unfavorable course leading to cardiopulmonary arrest, effectiveness of cardiopulmonary resuscitation measures greatly depends on an early start of such measures. In pregnant patients, not only rapid implementation of these measures is required, but a decision should also be taken about the convenience of fetal delivery through a perimortem Cesarean section.

**Case presentation:**

We report the case of thrombotic thrombocytopenic purpura in a 30-year-old primigravida white woman in week 28 of pregnancy that had a rapidly deteriorating course leading to cardiopulmonary arrest and an emergency perimortem Cesarean section resulting in fetal survival but maternal death. The patient was asymptomatic at admission and such an unfavorable evolution was initially unexpected. Analytical findings were treated with fresh frozen plasma and methylprednisolone but they did not improve. Plasmapheresis was considered but cardiac arrest rapidly ensued.

**Conclusions:**

Despite the low prevalence of thrombotic thrombocytopenic purpura, the finding in a pregnant woman of the triad consisting of anemia, thrombocytopenia, and neurological changes should guide clinical diagnosis, and should prompt measurement of the metalloprotease ADAMTS-13 in order to rule out or confirm diagnosis of thrombotic thrombocytopenic purpura and evaluate the best therapeutic option. If cardiopulmonary arrest occurs in a woman with a gestational age of more than 24 weeks, a perimortem Cesarean section is advised if the patient has not recovered her pulse after the first four minutes.

## Introduction

Thrombotic thrombocytopenic purpura (TTP) is, like hemolytic uremic syndrome (HUS), a type of occlusive thrombotic microangiopathy that is not specific to pregnancy, but occurs with an increased frequency during it [1]. The low prevalence of the condition and its severity increase the interest of reports on the outcome of diagnosed and treated cases, particularly during pregnancy. We report a case of TTP in a pregnant woman that had an unfavorable course, leading to cardiopulmonary arrest.

Despite the low overall frequency of TTP, there are two factors that have been related to its increased incidence rate during pregnancy: a hypercoagulable state, particularly in women with thrombophilia, and decreased levels of metalloprotease ADAMTS-13. Metalloprotease ADAMTS-13 is an enzyme responsible for binding to large multimers of von Willebrand factor (vWF), converting them into smaller molecules. These large vWF multimers may be seen in serum from patients with TTP due to decreased levels of ADAMTS-13, which causes an increased platelet adhesion along with those vWF multimers, leading to severe thrombocytopenia and microangiopathic hemolytic anemia associated with side effects in vital organs [2-4]. The reduced plasma ADAMTS-13 activity in familial TTP patients is usually a consequence of homozygous mutations in both of the ADAMTS-13 alleles located at chromosome 9 [5]. In acquired idiopathic TTP patients ADAMTS-13 activity is severely reduced only during an initial episode or later recurrences. Immunoglobulin G (IgG) autoantibodies that inhibit plasma ADAMTS-13 activity can be detected during acquired episodes in 44% to 94% of patients, suggesting the presence of a transient, or intermittently recurrent, defect of immune regulation [6].

Clinically, the triad of microangiopathic hemolytic anemia with schistocytes and a negative Coombs’ test, thrombocytopenia causing bleeding diathesis and fluctuating neurological changes is commonly seen in up to 75% of patients [7].

Prognosis of TTP has improved in the past 30 years with development of plasma replacement therapies based on administration of fresh plasma. Other treatments such as plasmapheresis (plasma exchange) help to remove large multimers and anti-ADAMTS-13 antibodies. These treatments achieve a satisfactory response in 90% of patients with TTP, with healing rates up to 80% [2-4].

## Case presentation

A 30-year-old primigravida Caucasian white woman in week 28 of pregnancy was referred to the emergency room of our hospital for dizziness in the past two days with normal blood pressure (BP) levels and episodes of diplopia, dyslalia, and paresthesia in the hands and lips, associated with a fronto-occipital headache. Prior progress of her pregnancy had been normal. The patient only reported that two weeks before she had noticed mild petechial lesions in her lower limbs, which she did not consider important, (she did not even report them at admission), together with some asthenia and adynamia. The last laboratory tests at week 25 had found a hemoglobin (Hb) level of 10.5g/dL and a hematocrit value of 30%. Hepatitis B virus, hepatitis C virus and human immunodeficiency virus test results were negative.

Upon arrival at our center, the patient was seen to have a marked skin, mucosal pallor and a good general condition, and she was oriented and cooperative. No edema was found. Her neurological examination was normal. She had a blood pressure (BP) value of 145/65mmHg. An ultrasound examination upon admission showed a fetus in a transverse position, with adequate biometrics (estimated weight, 1100g), and normal placenta and amniotic fluid. Her cardiotocographic recording was normal and there were no contractions.

Laboratory tests performed at admission found a hemoglobin level of 5.2g/dL, a platelet count of 5000, and a normal white blood cells (WBC) count. Her biochemical test results were as follows: lactic dehydrogenase (LDH) 1398U/L (control 87 to 241U/L), alkaline phosphatase 237U/L (control 50 to 135), serum glutamic-oxalacetic transaminase (S-GOT) 39U/L (control 15 to 37), serum glutamic-pyruvic transaminase (S-GPT) 62U/L (control 30 to 65), total bilirubin 4.2mg/dL (control <1mg/dL), and uric acid 4.7mg/dL (control 3.7 to 7.2). Her coagulation and kidney function were normal. As a first measure, transfusion was started of four units of a concentrate of red blood cells and two units of plasma (450cc). The patient was then admitted to the intensive care unit based on her laboratory findings, but without a clear diagnosis.

The patient maintained normal vital signs for eight hours following admission. The results of her laboratory tests after transfusion included: Hb 8g/dL, 21,000 platelets, LDH 1211U/L, S-GOT 74U/L, S-GPT 51U/L, total bilirubin 3.8mg/dL, and normal coagulation and kidney function. In a peripheral blood smear, the hematologist identified schistocytes (>8%) with an increased reticulocyte count, and diagnosis of thrombotic thrombocytopenic purpura was therefore suspected.

On that same morning, treatment was started with methylprednisolone, 60mg every 12 hours (2mg/kg/day) and fresh frozen plasma (225cc) every 12 hours. After repeat plasma administration in the evening, hematological control tests showed decreases in platelet count and hemoglobin. Her renal function remained normal, without proteinuria and her fluid balance was monitored.

Plasmapheresis was considered but, with no apparent cause, at 22:30h the patient experienced progressive breathlessness and moist rales that did not respond to oxygen therapy, morphine, and furosemide. The obstetric team was notified of the situation, and sedation and orotracheal intubation were concomitantly started. Exit through the tube of the pink foam characteristic of lung edema, hypoxia, and severe progressive bradycardia were seen, and cardiopulmonary arrest finally occurred.

Advanced cardiopulmonary resuscitation (CPR) procedures with chest compression and vasoactive drugs were started at 22:42h, while the obstetric team, after assessing the vital status of the fetus and because of the extreme severity of the mother, in cardiopulmonary arrest, decided to perform a perimortem emergency Cesarean section in the bed of the intensive care unit while resuscitation maneuvers continued. At 22:50h, a live fetus weighing 1173g with an Apgar score of 4 out of 6 was delivered by Cesarean section.

Advanced CPR procedures were continued for 45 minutes, including three defibrillations for ventricular fibrillation, until the patient finally experienced electromechanical dissociation and asystole, and she died at 23:37h.

**Figure 1:**
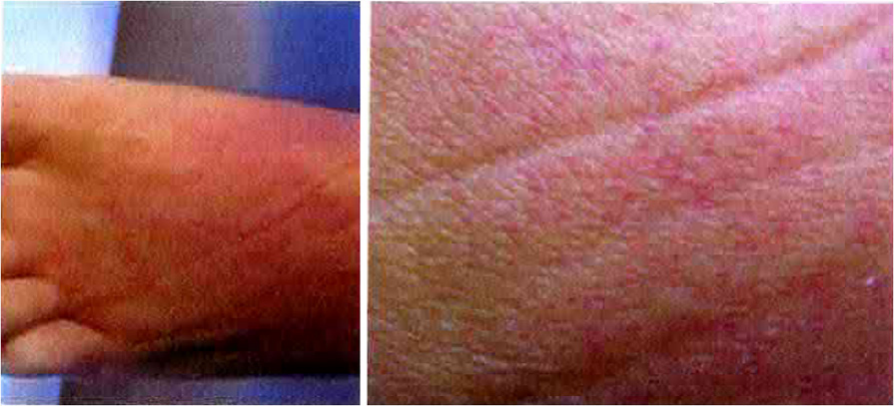
Autopsy: petechial lesions.

**Figure 2:**
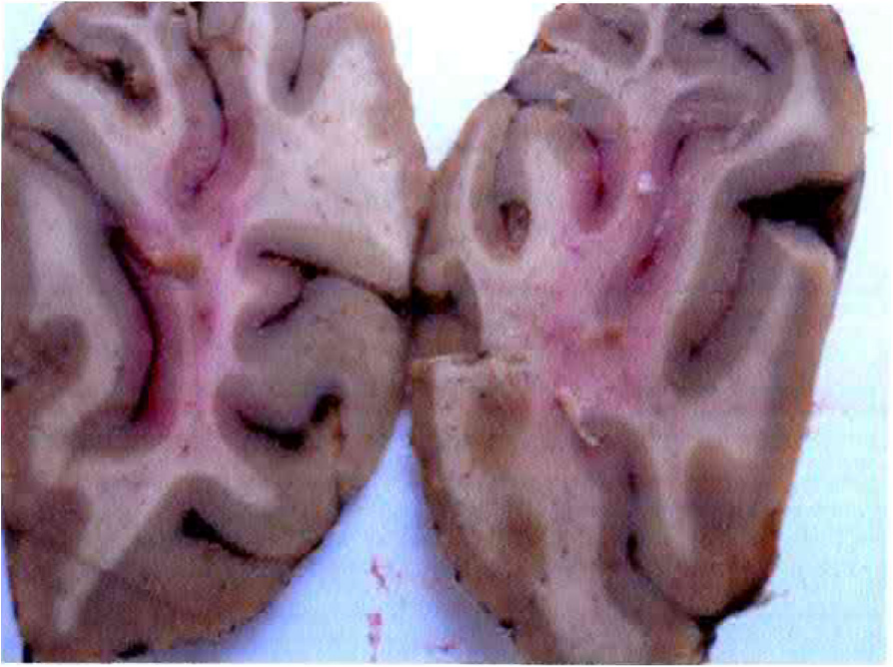
Autopsy: petechial lesions in the brain.

Autopsy findings included acute lung edema, multiple microthrombi, particularly in brain, heart, liver, pancreas and kidneys, consistent with a diagnosis of thrombotic thrombocytopenic purpura (Figures 1, 2).

## Discussion

There are two well-differentiated aspects in the reported case; on the one hand, the unfavorable course of TTP during pregnancy and, on the other hand, the management of cardiopulmonary arrest in a pregnant patient at the start of the third term of pregnancy.

In the reported patient, diagnosis of TTP was considered based on the laboratory findings, suggesting both hemolytic anemia and thrombocytopenia, which had not been suspected by her physician at the start of the episodes of neurological impairment.

The reported series show that the occurrence of evidence of microangiopathic hemolytic anemia, mild renal failure, severe thrombocytopenia (<25,000 platelets/microliter), and increased liver enzymes with an elevated LDH/GPT ratio in a pregnant woman with fluctuating neurological symptoms should suggest TTP as a first diagnostic hypothesis, rather than other potential microangiopathic conditions such as the HELLP syndrome [2-4].

When the patient was admitted to our hospital, the HELLP syndrome was one of the suspected diagnoses, but the patient only had a mild transaminase elevation at baseline. The patient had no elevated blood pressure levels at any time. On the other hand, unlike TTP, HUS occurs in the puerperal period in 90% of cases, mainly causes renal microangiopathy, and its most common clinical presentation includes edema, high blood pressure, hemorrhagic changes or severe renal failure, with diarrhea being a common close history.

Various studies have demonstrated an increased maternal and perinatal survival in cases of recurrent TTP as the result of regular monitoring. As patients have had prior episodes, the risk of recurrence is known.

Once diagnosis of TTP is suspected in a pregnant patient, it should be confirmed, and the need for performing treatment with plasma exchange or plasmapheresis to avoid worsening of the condition should be established. The benefit of a treatment protocol based on ADAMTS-13 levels has been reported [8-11].

The reported cases of pregnant women with recurrent or initial TTP show that measurement of the enzyme ADAMTS-13 allows for selecting which patients are amenable to medical treatment with corticosteroids and which would benefit from administration of plasma or plasmapheresis. As 20 to 50% of patients who survive the first episode experience a relapse one month or even years after the acute episode of TTP, some risk factors for recurrence have been analyzed. In these patients, the association of severe ADAMTS-13 deficiency and the presence of anti-ADAMTS-13 autoantibodies have been reported as the most negative prognostic markers at remission, increasing the relative risk of TTP recurrence by 3.6 times [12,13]. In order to prevent the recurrence of TTP, ADAMTS-13 levels and anti-ADAMTS-13 autoantibodies levels should be assessed in pregnant women with prior episodes. The use of the monoclonal antibody rituximab has been successfully reported in relapsing TTP patients as a therapeutic option [14].

The lack of an initial clear diagnosis and the sudden progress of the clinical condition did not allow for measurement of ADAMTS-13 levels in our case.

TTP is a condition with a high mortality rate whose prognosis largely depends on early diagnosis and treatment. As delivery does not generally cause resolution of TTP, it is not routinely indicated, especially under 34 weeks, although it may be required if TTP is associated with preeclampsia [9]. We do not know if we would have been able to achieve a better response to treatment with corticosteroids and plasma after fetus delivery in the reported case. Our approach was initially conservative because of the risk of prematurity associated to such a short gestational age (28 weeks).

In the reported case, diagnosis was made six hours after admission to our hospital, and treatment was started with corticosteroids and plasma. After a slight initial improvement, the patient evolved in only eight hours to a tragic, irreversible condition that led to massive lung edema and cardiac arrest.

Although management of cardiac arrest is always complex, such complexity is much greater when this occurs in a pregnant woman at the end of the second term or during the third term of pregnancy because the life of the fetus is also in danger. The effectiveness of cardiopulmonary resuscitation measures greatly depends on their early implementation. In a pregnant patient, not only rapid implementation of such measures is required, but a decision should be taken about the convenience of fetus delivery. In the case reported, the fetus was delivered seven minutes after the start of cardiopulmonary resuscitation maneuvers. Two of those seven minutes were required for traveling to the intensive care unit, four minutes to evaluate fetal well-being, confirm the lack of maternal response to resuscitation maneuvers and decide the conduct of the Cesarean section, and one minute for fetal delivery. The convenience of achieving fetal delivery in accordance to the ‘four-minute rule’ is based on the need to prevent the irreversible damage to fetal brain that may occur when placental blood flow is stopped for longer than five minutes. If resuscitation maneuvers fail to recover a maternal pulse in four minutes, a perimortem Cesarean section is indicated [15,16]. Two years later, the child of the reported patient is showing normal cognitive development.

## Conclusions

Despite the low prevalence of TTP, the finding in a pregnant woman of the triad consisting of anemia, thrombocytopenia, and neurological changes should prompt measurement of the metalloprotease ADAMTS-13 in order to rule out or confirm diagnosis of TTP and evaluate the best therapeutic option. If cardiopulmonary arrest occurs in a woman with a gestational age of more than 24 weeks, a perimortem Cesarean section is advised if the patient has not recovered her pulse after the first four minutes.

## Consent

Written informed consent was obtained from the patient’s family for publication of this case report and any accompanying images. A copy of the written consent is available for review by the Editor-in-Chief of this journal.

## Competing interest

The authors declare that they have no competing interests.

## Authors’ contributions

EGM and IN attended the patient and were the major contributors in writing the manuscript. IC also participated in patient attendance and contributed in writing the clinical case. MB performed the bibliographic search and wrote the introduction. All authors read and approved the final manuscript.
